# Different Magnitudes of Tensile Strain Induce Human Osteoblasts Differentiation Associated with the Activation of ERK1/2 Phosphorylation

**DOI:** 10.3390/ijms9122322

**Published:** 2008-11-26

**Authors:** Junfeng Zhu, Xiaoling Zhang, Chengtao Wang, Xiaochun Peng, Xianlong Zhang

**Affiliations:** 1 Department of Orthopaedics, Shanghai the 6th People’s Hospital, Jiaotong University, Shanghai, 200233, China. E-Mails: zhujf2002@hotmail.com (J. Z.); xcpeng81@163.com (X. P.); 2 Institute of Health Science, Shanghai Institute for Biological Sciences, Chinese Academy of Sciences, Shanghai, 200025, China. E-Mail: xlzhang@sibs.ac.cn; 3 School of Mechanical Engineering, Shanghai Jiaotong University, Shanghai, 200030, China. E-Mail: ctwang@sjtu.edu.cn

**Keywords:** Prosthesis, Strain, Finite element, Osteoblast, Differentiation

## Abstract

Mechanical factors are related to periprosthetic osseointegration following total hip arthroplasty. However, osteoblast response to strain in implanted femurs is unclear because of the absence of accurate stress-measuring methods. In our study, finite element analysis was performed to calculate strain distribution in implanted femurs. 0.8-3.2% tensile strain was then applied to human osteoblasts. Higher magnitudes of strain enhanced the expression of osteocalcin, type I collagen, and Cbfa1/Runx2. Lower magnitudes significantly increased ALP activity. Among these, type I collagen expression increased with the activation of ERK1/2 phosphorylation in a strain-magnitude-dependent manner. Our study marks the first investigation of osteoblast response at different magnitudes of periprosthetic strain. The results indicate that the functional status of human osteoblasts is determined by strain magnitude. The strain distribution in the proximal region of implanted femur should be improved for osseointegration.

## 1. Introduction

Periprosthetic osseointegration following total hip arthroplasty (THA) plays a key role in the stability of cementless stems. Bone formation around prostheses has been investigated at tissue level both in animal experiments and clinical radiological follow-up. At a cellular level, osteoblasts, which are cells close to the bone-implant interface, play an important role in the postoperative osseointegration of stems by producing the mineralized matrix. Various mechanical stimulations such as stretch influenced the proliferation and differentiation of osteoblasts *in vitro* [1–2]. Although it has been reported that stress magnitude may contribute to the way cells respond to mechanical stimulation [3–4], the response to periprosthetic strain in implanted femurs is unclear because of the absence of accurate stress-measuring methods. Recently, finite element analysis (FEA) has been a powerful tool used frequently to calculate the tissue strains in biomechanical studies [5]. Unfortunately, among these, there is limited research on the mechanical response of osteoblasts. In our study, a CT-based finite element model of the implanted human femur was built for analyzing the tissue-level strain magnitude at the bone-implant interface. The results of FEA were amplified 20 times to stimulate human osteoblasts *in vitro*. Our study marks the first investigation of osteoblast response at different magnitudes of periprosthetic strain. Given that osteoblast is the only cell in body that is capable of producing new bone, the information on strain sensitivity of osteoblasts may provide clues for speculating the status of periprosthetic osseointegration. Decreased periprosthetic osseointegration as well as osteoclast-mediated resorption was recognized as an important factor influencing implant survival time. Therefore, the information from this study may contribute to the development of a strategy for reducing the risk of prosthesis loosening.

## 2. Results and Discussion

**Figure 1.**
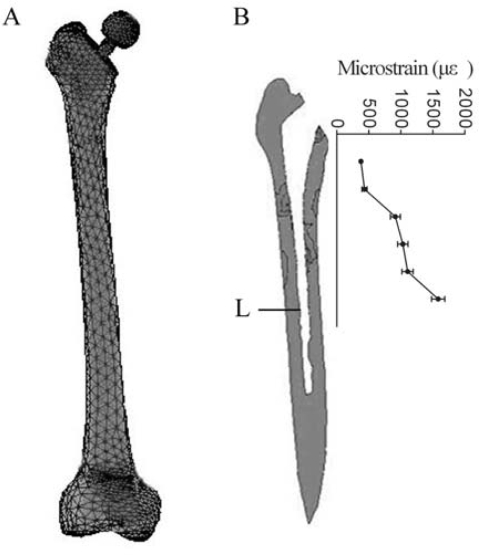
The tissue-level strain magnitude at the bone-implant interface **(A)** A three-dimensional finite element (FE) model of the implanted human femur. 29,841 elements were generated in the FE model, including 4,193 elements at the bone-stem interface. **(B)** The strain distribution at the bone-implant interface. From 375 μɛ to 1,583 μɛ, the interface bone strain increased along the stem axis. A peak strain of 1,583 μɛ was found near the prosthetic tip. L: the level of the prosthetic tip. The white space below the prosthetic tip is the medullary cavity.

Tissue-level strains in intact human bone are usually less than 1000 μɛ [6]. After the insertion of an untapered femoral prosthesis, a peak strain of 1868 μɛ was reported near the tip [7]. Figure 1A shows 29,841 elements in the THA finite element model, including 4,193 elements at the bone-implant interface. Owing to the improvement of prosthetic shape, a lesser peak strain of 1583 μɛ was found in our study. From 375 μɛ to 1583 μɛ, the interface bone strain increased along the stem axis. (Figure 1B) Similar strain pattern was stated in measuring the surface strains of the implanted femur [8]. We focused on the response of human osteoblast to different magnitudes of strain at the bone-implant interface, which was important for the postoperative stability of cementless stems. However, most investigations have demonstrated that the magnitude of tissue-level strain was too low to induce cell response. The reason is that the strain loaded on cells inside the bone is different from the tissue-level strain calculated by FEA. There has been a strain amplification mechanism on osteoblasts inside the bone which mainly caused by the fluid flow around the osteoblasts [9]. Recently, a quantitative strain model at cellular level stated that strains loaded on the whole bone might be amplified over 20 times at the membrane of osteoblasts [10]. The amplification factor was calculated as a function of the load frequency and extensively justified to induce effective mechanical response of cells. For example, 0.3–5% tensile strain was produced according to the quantitative model and significantly induced the expression of osteoblast-specific genes in osteoblasts [10–12]. Based on the information, the strain magnitude at tissue level can be converted to the corresponding magnitude at cellular level. Here, the tissue-level strain of 375μɛ∼1583μɛ was amplified 20 times to stimulate human osteoblasts *in vitro*.

**Figure 2.**
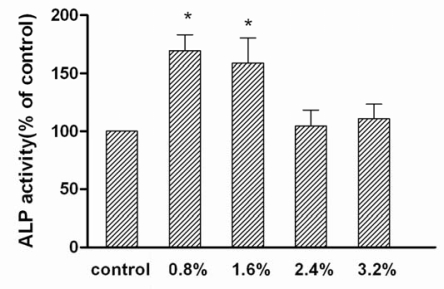
Lower magnitudes of tensile strain increased ALP activity. ALP activity increased at 0.8% and 1.6% elongation, but remained unchanged at higher magnitudes of strain. ALP activity per sample was normalized for the total protein content of the cell lysate. Results are presented as the percentage of activity change as compared to the control group. “*” indicates P < 0. 01.

**Figure 3.**
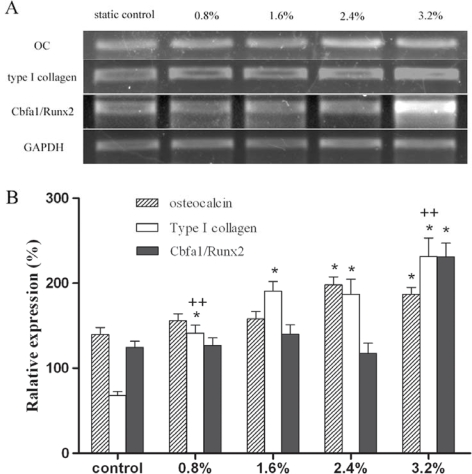
Higher magnitudes of tensile strain induced the expression of osteocalcin (OC), type I collagen and Cbfa1/Runx2 in human osteoblasts. A. Agarose gel electrophoresis of PCR products. B. Semiquantitative results of osteocalcin, type I collagen, and Cbfa1/Runx2 gene normalized for GAPDH expression. P < 0.01, * as compared to static control, ++ as compared to the group with 1.6% elongation.

ALP activity, expression of type I collagen and Cbfa1/Runx2 were suggested to be associated with the magnitude of strain [12, 13]. ALP activity and osteocalcin are considered as the indicators of osteogenesis. In bone marrow stromal cells, ALP activity increased at 5% elongation, then decreased at 10% and 15% elongation [13]. Figure 2 showed that the ALP activity of human osteoblasts increased at 0.8% and 1.6% elongation, but remained unchanged at higher magnitudes of strain. In contrast, higher magnitudes of strain (2.4% and 3.2%) played a positive role in the expression of osteocalcin, while 0.8% and 1.6% elongation had no effects (Figure 3). Synthesized and secreted exclusively by osteoblasts at the late stages of maturation, osteocalcin regulates bone growth by binding to hydroxyapatite crystals, the key mineral component of bone [14]. At the mineralization stage, ALP activity decreased, but the expression of hydroxyapatite deposition-related genes such as osteocalcin increased in osteoblasts. Therefore, higher magnitudes of strain could further promote the functional status of osteoblasts by enhancing extracellular matrix maturation. This finding suggests that the osteoblasts around prosthetic tip may show more active functional status than those around the proximal part of prosthesis, since the interface bone strain increased along the stem axis in FEA model. Based on the foregoing results of FEA, the osteoblasts around the proximal part of prosthesis might be inhibited at the early stage of differentiation. This difference may lead to decreased bone formation around the proximal part of prosthesis. The dual characters of osteoblasts in response to mechanical strain were different from their progenitor cells. Those cells mostly showed an increase of ALP activity and responded to a broader magnitude spectrum. For example, 0.8% and 5% elongation both significantly increased the ALP activity of bone marrow stromal cells, but this had no effects on the expression of osteocalcin [13]. The differential regulation of ALP activity and osteocalcin expression might be attributed to the differential activation of various signaling pathways including cytoskeletal integrin rearrangement, stretch-activation of cation channels, and mitogen-activated protein kinases (MAPK) family.

Type I collagen accounts for 90% of bone matrix proteins [15]. Most investigations have demonstrated that mechanical strain stimulated the expression of type I collagen [12, 13]. Differential responses were observed in our study. As compared to static control, the mRNA level of type I collagen increased in a strain-magnitude-dependent manner (Figure 3). The increasing strain gradually enhanced the expression of type I collagen in human osteoblast. As the initial step in bone tissue formation, the synthesis of type I collagen provides organic scaffold for the subsequent deposition of mineral. New bone mass can only be acquired by increased matrix synthesis [16]. In term of this viewpoint, higher magnitudes of strain would enhance periprosthetic bone formation by initiating the increase of type I collagen synthesis in human osteoblasts. Associated with the FEA result of the THA model, the expression of type I collagen was consistent with a postoperative histological phenomenon that hyperostosis was observed near the tip of the untapered stem where postoperative strain distribution markedly increased. Meanwhile, bone loss took place around the proximal part of femoral prosthesis where strain magnitude robustly decreased. All these evidences indicate that lower magnitudes of strain which are mainly located around the proximal part of prosthesis will lead to decreased bone formation compared with that of higher magnitudes; this may be an important factor influencing implant survival time. Therefore, the adverse strain distribution in the proximal region of implanted femurs should be targeted for reducing the risk of prosthesis loosening. Many FEA models showed that the strain distribution in implanted femurs is related to the stiffness and shape of the prosthesis.

The use of a lower modulus or composite femoral stem might allow better load sharing and ultimately achieve a more stable biomechanical harmony [17]. Owing to excessive interfacial micromotion, the clinical results of this kind of system such as a Proplast-coated prosthesis were unsatisfactory in previous investigations. However, in term of the osteoblast response in this study, the lower-modulus femoral component might still have potential advantage if its interfacial micromotion could be improved. Cbfa1/Runx2 is a key transcription factor which regulates osteoblasts differentiation. As the end point of the MAPK signaling pathway, Cbfa1/Runx2 regulated the expression of osteoblast-specific genes after binding to osteoblast-specific cis-acting element [18]. Mechanical strain could increase [13, 18] or decrease [19] the Cbfa1/Runx2 mRNA level. Figure 3 shows that the Cbfa1/Runx2 mRNA increased only at the highest magnitude of strain in human osteoblasts. At 3.2% elongation, mechanical strain plays a positive role in the expression of osteocalcin and type I collagen. However, the expression of these genes and ALP activity also increased at other lower magnitudes, while the expression of Cbfa1/Runx2 remained unchanged (Figures 2, 3). Similarly, the absence of Cbfa1/Runx2 expression was observed at protein level (data not shown). This phenomenon was not consistent with the foregoing “end point” theory on MAPK signaling pathway. The results suggest the presence of mechano-transduction threshold in the stretch-induced expression of Cbfa1/Runx2 and indicate that Cbfa1/Runx2 may not be indispensable for the expression of osteoblast-specific genes. A crosstalk pathway and other principal regulators might be involved in the mechanical signal transduction.

**Figure 4.**
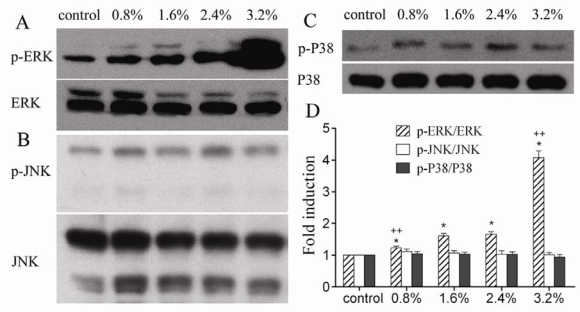
The effect of different magnitudes of strain on MAPK activity. Western blot of MAPK was shown as A. p-ERK and ERK, B. p-JNK and JNK, and C. p-p38 and p38. ERK1/2 was phosphorylated in a load-dose-dependant manner, whereas JNK and P38 were unaffected. D. Quantification graph for the protein levels of the phosphorylated kinases. The results were normalized to total kinase levels and expressed as a ratio to corresponding static control. P < 0.01, * as compared to the static control, ++ as compared to the group with 1.6% elongation.

As MAPK family members, extracellular signal-regulated kinases (ERK1/2), c-jun N-terminal kinase (JNK), and p38MAPK were activated upon mechanical stimulation of the cells in several studies [16, 20, 21]. However, the reported phosphorylation of these members widely differed. Figure 4 shows that ERK1/2 was phosphorylated in a load-dose-dependant manner, whereas JNK and P38 were unaffected. This result was different from previous report that only JNK was activated by 9% tensile force applied in human osteoblasts [20]. All these evidences indicate that the selective activation of MAPK family members is related to the strain magnitude. This finding might justify the differential responses of human osteoblasts at different magnitudes of periprosthetic strain. It appears that the strain magnitudes used in our study were excluded from the magnitude spectrum for activation of JNK and P38 phosphorylation. The results showed that the increased expression of osteoblast-specific genes in human osteoblasts was associated with the activation of ERK1/2 phosphorylation. Interestingly, the activation of ERK1/2 phosphorylation in our study was highly consistent with the stretch-induced expression of type I collagen.(Figure 3B, 4D) Whether ERK1/2 signaling pathway plays a leading role in the mechanical induction of collagen gene requires further investigation. If it is true, the ERK1/2 signaling pathway might be a potential target for improving the overall integration of prosthesis.

## 3. Experimental Section

### 3.1. Finite element (FE) model

FE models of European femora have been extensively investigated. However, significant dimensional differences existed between the proximal femora of Asians and Caucasians [22, 23]. Smaller offset, narrower canal especially at the isthmus, and differential endosteal shape were found in Chinese [24, 25]. In term of the ethnicity and skeletal characteristics of our target patients, a Chinese human femur was selected from 120 embalmed specimens, which was considered average in shape and bone density. The placement of a cementless titanium-alloy stem was performed according to the surgical manual. The CT scan images of the model were transferred to a graphics computer program. Based on the contours of the cortical bone, cancellous bone, and prosthesis, a three-dimensional FE model was constructed with a 10-node tetrahedral element. CONTA174 and TARGE170 elements were used to model the contact interface. The titanium stem and bone were assumed to be isotropic and linear elastic. The Poisson’s ratios for the stem and bone were set to be 0.3. Young’s modulus of 107 GPa and 17 GPa [10] were assigned to the stem and cortical bone. Young’s modulus of the cancellous bone was linearly converted from CT Hounsfield values [26, 27]. The joint load of 1631N and muscle forces were applied based on published data [28, 29]. The strain distribution at the bone-implant interface was analyzed using FE software ANSYS 5.7.

### 3.2. Cell culture

SV40 human osteoblasts (sv40hOB, ATCC) were obtained from the Shanghai Institutes for Biological Sciences, Chinese Academy of Science, P.R. China. The mechanical sensitivity of sv40hOB cells was verified in previous studies [2, 30]. Cells were seeded to six-well BioFlex culture plates (Flexcell International, Hillsborough, NC, USA) with flexible membranes coated with collagen I, at a density of 3.0 × 10^4^ cells/cm^2^. They were cultured in DMEM/F12 medium (2 mL) supplemented with G418 (0.3 mg/mL), ascorbic acid (50 mg/L), 10 mM β-glycerophosphate (Sigma, St. Louis, MO, USA) and 10% fetal bovine serum (HyClone, USA). Finally, they were incubated under 5% CO_2_ atmosphere at 37 °C for four days to reach 90% confluence. The medium was changed every two days.

### 3.3. Application of mechanical strain

Radial and circumferential strain was provided by an FX-4000T Flexcell BioFlex Tension Plus Unit (Flexcell International, USA). BioFlex culture plates were placed on a 25 mm diameter loading station. When vacuum pressure was applied to the plates through a vacuum pump, the membrane was deformed to create regulated strain. After reaching 90% confluence, the cells were respectively subjected to tensile strains of 0.8% (7,500 μɛ), 1.6% (16,000 μɛ), 2.4% (24,000 μɛ), and 3.2% (31,660 μɛ) for 48h. Static controls were not stretched. One-half sine wave and a work frequency of 1 Hz were selected in this experiment.

### 3.4. Alkaline phosphatase (ALP) activity assay

ALP activity was assessed as described by Kasten [31]. The supernatant was incubated at 37 °C for 15min with p-nitrophenyl phosphate. The coloring reaction was measured at 405 nm. Protein concentration was determined with a bicinchoninic acid (BCA) protein assay kit (Pierce Chemical, USA). ALP activity per sample was normalized to the amount of total protein and displayed relative to the control.

### 3.5. Semi-quantitative RT-PCR

After being stretched, total RNA was extracted from the cells by using TRIzol reagent (Invitrogen, USA) according to the manufacturer’s instructions. Then 1 μg RNA was reverse-transcribed for first strand cDNA synthesis (RevertAidTM M-MuLV, Fermentas, USA). PCR amplification was performed using the primers listed in Table 1. RT-PCR products were electrophoresed on 1% agarose gel with 0.5 mg/mL ethidium bromide. Bands were detected by UV illumination of ethidium bromide-stained gels. Band intensities were quantitatively analyzed by Quantity One software for each gene and were normalized to the corresponding GAPDH values.

### 3.6. Western blot analysis of MAPK

After being stretched, the cells were rinsed with PBS and lysed in SDS sample buffer (0.2 mL; 62.5 mM Tris-HCl, pH 6.8, 2% SDS, 10% glycerol, 50 mM DTT, and 0.1% bromphenol blue). The samples were kept on ice and then boiled for 5 min. Cells lysates were separated by 10% SDS-polyacrylamide gel electrophoresis and electrotransferred to polyvinylidene difluoride membranes (Millipore, USA). After being blocked with 5% skim milk for 2 h at room temperature, the membrane was probed overnight at 4°C with anti-ERK, anti-JNK, anti-p38, anti-phospho-p38, anti-phospho- ERK, and anti-phospho-JNK(Cell Signaling, USA), respectively. After extensive washing, the membranes were incubated for 1 h at room temperature with an anti-rabbit secondary antibody conjugated to horseradish peroxidase. Immunoreactive bands were detected by an enhanced chemiluminescence system (Pierce) followed by exposure to X-OMAT Kodak films. The density of the bands was analyzed, and the results were normalized to total ERK1/2, JNK, and p38 MAPK.

### 3.7. Statistical analysis

All assays were repeated in two independent experiments with a minimum of n = 3 for each data point. Statistical analysis among groups was performed by ANOVA and SNK test using SAS6.12 software package (SAS Ltd., NC, USA). Statistically significant values were defined as *P* < 0.05.

## 4. Conclusions

The stretch-induced expression of osteoblast-specific genes in human osteoblasts is dependent on strain magnitude and associated with the activation of ERK1/2 phosphorylation. Higher magnitudes of periprosthetic strain increased the mRNA levels of osteocalcin, Cbfa1/Runx2, and type I collagen; this might owe to the differential level of ERK1/2 phosphorylation. Based on the results of FEA, lower magnitudes of strain which are mainly located around the proximal part of prosthesis compromise the functional status of human osteoblasts and contribute to decreased bone formation around the proximal part of prosthesis. The decreased periprosthetic osseointegration will make space for wear particles and lead to aseptic prosthesis loosening following THA. Based on this finding, the use of a lower modulus or composite femoral stem might still have potential advantage if its interfacial micromotion could be improved. In addition, a potential crosstalk pathway and some principal regulators need to be further investigated. If the vital role of ERK1/2 phosphorylation in the mechanical response of human osteoblasts can be further supported by more preclinical trials, a special biological target may be proposed for reducing the risk of prosthesis loosening following THA.

## Figures and Tables

**Table 1.** Primer sequences and cycle conditions used for RT-PCR.

Gene	Primer sequence(Forward/Reverse)	T annealing	Cycles
GAPDH	5′ GTTCCAATATGATTCCACCC 3′	52°C	21
	5′ AGGGATGATGTTCTGGAGAG 3′		
Type I collagen	5′ ACAGCCGCTTCACCTACAGC 3′	52°C	22
	5′ TGCACTTTTGGTTTTTGGTCAT 3′		
Osteocalcin	5′ GCCTTTGTGTCCAAGC 3′	51°C	30
	5′ GGACCCCACATCCATAG 3′		
Cbfa1/Runx2	5′ TACCTGAGCCAGATGACG 3′	58°C	28
	5′CAGTGAGGGATGAAATGC3′		
